# Race Progress and the Declining Birth-Rate

**Published:** 1907-10-05

**Authors:** 


### RACE PROGRESS AND THE DECLINING BIRTH-RATE.

In a recent report on infantile mortality the Medical Officer of Health for Manchester, Dr. Niven, touches on an aspect of the efforts now being made to reduce the great waste of infant life which is probably destined to grow into one of greater importance. He says:—

> We are in the presence of a movement of suppression of reproduction. Every step taken requires to be considered as to its probable effects on this movement. A continuance in the decline of the birth-rate must be disastrous to the nation. It is difficult to see what forces are to be opposed to it. What is wanted primarily is a higher standard of life, a higher standard of education for all, in which the brain and hand are alike trained to their fullest power of execution and enjoyment.

A higher standard of life and education for all will certainly act in the way of lessening the great waste of infant life, but we doubt seriously if it will tend to lessen the movement of suppression of reproduction.

On the contrary, it is extremely probable that this movement is the outcome of the higher standard of life already attained. It is probably in large measure an expression of prudence in the assumption of parental responsibility rendered necessary by the higher standard of life which has to be maintained for each member of the family within the economic limitations imposed by the family income. Certainly it is in the higher social strata of the nation that the phenomenon of diminished reproductivity is most to be observed. Such statistics as are available show that among the earning classes it is in the higher branches that the lowest birth-rates prevail, while among the lower orders of the working classes the birth-rate remains undiminished, and continues at a rate far in excess of the provision which the parents are able to make for their numerous offspring. And this is why the declining national fertility bears so serious an aspect. A mere quantitative estimate of human fertility is no measure of the progress or decline of a people in world achievement. It is obvious that excessive reproduction in our congenital criminal or imbecile classes is as calamitous as the sterility of the higher and more desirable types in society.

These are extreme examples of what appears to be happening in England to-day. The people who are achieving the higher standard of life at which we aim are those whose declining fertility is manifesting itself in our diminished birth-rate. Were the reverse the case, were it that the degraded elements in our population are ceasing to people the earth, and that to this cause is due the decline in our birth-rate we confess we should look upon the decline without misgiving. But the effect of a lessened fertility of the more desirable elements in our population, coupled with an undiminished fertility of the less desirable, is bound to react detrimentally upon racial progress. France was the first nation in Europe to show a steadily and rapidly declining birth-rate, England is following in her wake. Since 1876 the decline in the English birth-rate has been practically uninterrupted, and it has fallen since then from 36.3 per 1,000 persons living to 27.2 per 1,000 in 1905. In 1860 the Berlin birth-rate was 206.8 per 1,000 married women; in 1867 it was 224.7; in 1876 it was 240.3. Just as in this country, the Berlin birth-rate reached its maximum in the year 1876. It has since continued to decline. By 1885 it had fallen to 179.4 per 1,000 married women, by 1895 to 138.5, and by 1905 to 109.7. It is always consoling to have companions in misfortune, and if misfortune it be, it is certainly robbed of some of its terrors in that it is shared by international rivals.

